# Influence of UAV Flight Pattern on Droplet Deposition in Citrus Canopies

**DOI:** 10.3390/plants15172621

**Published:** 2026-08-27

**Authors:** Yifang Han, Yipeng Zhang, Yilong Zhan, Jinsheng Huang, Yingdong Qin, Jun Cai, Yubin Lan

**Affiliations:** 1 College of Information Science and Technology, Jinan University, Guangzhou 510632, China; hanyifangfangfang@163.com; 2 School of Cyber Security, Guangdong Polytechnic Normal University, Guangzhou 510665, China; 3 College of Artificial Intelligence and Low-Attitude Technology, South China Agricultural University, Guangzhou 510642, China; zhangyipeng@stu.scau.edu.cn (Y.Z.); zylong@scau.edu.cn (Y.Z.); huangjinsheng@stu.scau.edu.cn (J.H.); qinyingd@foxmail.com (Y.Q.); 4 Department of Biological and Agricultural Engineering, Texas A&M University, College Station, TX 77845, USA; 5 Texas A&M AgriLife Research and Extension Center, Beaumont, TX 77713, USA

**Keywords:** UAV, citrus, flight pattern, droplet deposition, canopy penetration rate

## Abstract

Pests and diseases can lead to a decline in citrus fruit quality and yield. Unmanned Aerial Vehicle (UAV) spraying provides rapid field operation and strong adaptability to complex terrain, and low-altitude, low-volume spraying for orchard pest management. However, existing studies have not yet fully clarified how different UAV flight patterns affect droplet deposition within citrus canopies and the surrounding space. This study evaluated two flight patterns using an XAG P20 UAV: flight pattern 1, parallel to the citrus tree rows, and flight pattern 2, perpendicular to the tree rows. Rhodamine B was used as a tracer, and Mylar cards were placed in the upper, lower outer, and lower inner canopy layers and on sampling rods around the trees. Total canopy deposition, deposition uniformity, canopy penetration, and rod-position deposition were analyzed. Flight pattern significantly affected total canopy deposition (*F* = 28.486, *p* = 0.0059). Compared with flight pattern 1, flight pattern 2 increased total canopy deposition by 72.19%. Deposition in the upper and lower inner layers increased by 88.40% and 94.31%, respectively, while canopy penetration increased from 28.23% to 34.15%. For both flight patterns, deposition on sampling rods located directly beneath the UAV flight path was significantly higher than that on rods located away from the flight path, by approximately 6.8 and 6.4 times for Patterns 1 and 2, respectively. Overall, flying perpendicular to the citrus tree rows improved total canopy deposition, inner-canopy deposition, and canopy penetration. This study provides a reference for optimizing UAV spraying patterns in citrus orchards and improving effective droplet deposition within the canopy, thereby contributing to safeguarding citrus yield and fruit quality.

## 1. Introduction

Citrus (*Citrus reticulata* Blanco) is one of the most important economic crops worldwide and is the largest fruit crop in terms of global production. It is also one of the most extensively cultivated and economically important fruit trees in southern China [1]. In 2007, both the citrus-growing area and citrus production in China surpassed those of Brazil, making China the world’s largest citrus-producing country [2,3]. With the continuous expansion of citrus cultivation, the occurrence of orchard pests and diseases has become increasingly serious, posing substantial challenges to the stability and sustainable development of the citrus industry [4,5]. Therefore, effective pest and disease control is of great importance to citrus production.

At present, pest and disease control in orchards still relies mainly on manual spraying and ground-based mechanical spraying [6]. Although manual spraying provides high operational flexibility, it is characterized by high labor intensity, low efficiency, poor application uniformity, and a high risk of pesticide exposure for operators [7,8]. Ground-based sprayers have a relatively large payload and stable spraying capacity; however, their mobility and operational adaptability are considerably restricted in hilly and mountainous orchards [9,10]. Citrus orchards in southern China are widely distributed in hilly areas, where inter-row access is often limited and citrus canopies are dense. Under such conditions, conventional spraying methods have difficulty simultaneously ensuring high operational efficiency, uniform deposition, and effective canopy penetration. UAVs offer the advantages of low-altitude and low-volume spraying, high operational efficiency, strong terrain adaptability, and improved operator safety. They can reduce the constraints imposed by complex terrain and have gradually become important equipment for pest and disease control in orchards [11,12,13,14]. It should be noted that regulatory policies on aerial pesticide application vary considerably among countries and regions. In the European Union, Directive 2009/128/EC on the sustainable use of pesticides generally prohibits aerial spraying in order to reduce potential risks to human health and the environment, although derogations may be granted under specific conditions in some Member States. Available statistics indicate that, in 2015, at least nine EU Member States approved such derogations, covering more than 450,000 ha. In 2019, aerial pesticide application in Europe was mainly concentrated in Spain and Hungary. Therefore, the practical adoption of UAV spraying should consider not only application performance but also compliance with regional regulations governing aerial pesticide application and pesticide use.

With the increasing application of UAV in orchard spraying, extensive research has been conducted on the optimization of operational parameters and spraying systems. Previous studies have shown that flight height, flight speed, nozzle flow rate, spray volume, droplet size, nozzle type, and nozzle arrangement can all affect droplet deposition, coverage, drift loss, and canopy penetration [15,16,17,18]. Chen et al. optimized the operating height, flight speed, and nozzle flow rate of UAV in a citrus orchard through orthogonal experiments and found that different combinations of operational parameters significantly affected canopy droplet deposition [15]. Meng et al. compared the effects of flight route, flight speed, nozzle flow rate, and number of spray passes on droplet coverage in peach canopies and reported that tree architecture influenced the selection of an appropriate flight route [19]. In addition, Xu et al. showed that rotor airflow is an important factor affecting droplet movement and deposition during UAV spraying, and that changes in rotor airflow intensity and spatial distribution directly influence droplet velocity, dispersion range, and deposition performance [20,21,22]. From the perspective of droplet transport and deposition mechanisms, sprayed droplets are subjected to multiple effects, including rotor-induced downwash airflow, gravity, and target interception. Their trajectories and final deposition behavior are therefore closely related to the local flow field and target structure. Previous multiphase-flow studies have shown that external flow conditions, particle inertia, and interfacial interactions can significantly affect the transport and deposition behavior of droplets or particles [23,24].

In orchard UAV spraying research, increasing attention has been paid to the matching relationship between flight patterns and canopy spatial structure. Compared with field crops such as rice and wheat, fruit trees have larger canopy volumes, distinct gaps between trees and rows, and more complex canopy structures, which impose greater requirements on flight-route planning [25]. Previous studies have compared the effects of different UAV flight patterns on droplet deposition in vineyards, peach orchards, apple orchards, and pear orchards. The results showed that flight pattern can alter droplet distribution among vertical canopy layers, between inner and outer canopy zones, and in off-target areas [26,27,28]. Guo et al. compared five flight patterns in a mountainous Nanguo pear orchard and found that different flight patterns changed the vertical distribution of droplets and the deposition patterns between the inner and outer canopy zones. They further suggested that flight patterns should be selected flexibly according to operational objectives and orchard conditions [8]. These studies indicate that flight pattern not only affects the organization of UAV operations, but also changes the spatial overlap between the spray swath and the canopy, thereby influencing droplet deposition efficiency and canopy penetration.

However, most existing studies have focused on flight height, flight speed, nozzle parameters, spraying systems, and tree architecture, whereas the spatial relationship between flight pattern and tree-row direction remains insufficiently investigated. In citrus orchards, fruit trees are generally planted in rows, and the upper, outer, and inner canopy regions differ in their ability to intercept droplets. When the UAV flight direction changes from parallel to perpendicular to the tree rows, the spatial overlap between the spray release zone and the discrete tree canopies also changes. Meanwhile, the interaction between rotor-induced airflow and the heterogeneous spatial boundaries formed by the “canopy–inter-tree gap–inter-row gap” structure may also be altered. However, it remains unclear how this spatial relationship between flight direction and tree-row orientation jointly affects the redistribution of droplets within the upper canopy and inner canopy regions, and the space surrounding the trees, and experimental analyses integrating canopy deposition with spatial deposition around the flight path are still limited. Therefore, further investigation of droplet deposition characteristics under different flight directions from the perspective of spatial matching between flight paths and tree canopies is warranted.

Based on the above issues, this study focused on a regularly planted citrus orchard and evaluated two plant-protection UAV flight patterns: Pattern 1, in which the UAV flew parallel to the citrus tree rows, and Pattern 2, in which the UAV flew perpendicular to the tree rows. The study primarily investigated how the spatial relationship between UAV flight direction and tree-row orientation affected droplet deposition distribution. It should be emphasized that the present study focuses on the relative differences in droplet deposition between two UAV flight patterns, rather than on evaluating the overall plant-protection efficiency of UAV spraying or comparing UAV application with conventional orchard sprayers. By analyzing tracer deposition in different canopy regions and at locations surrounding the trees, differences in overall canopy deposition, internal-canopy deposition, and flight-path-related spatial deposition under the two flight patterns were quantified, thereby clarifying the effects of UAV flight pattern on droplet deposition in citrus trees. The results provide a reference for optimizing UAV spraying patterns and improving precision pesticide application in citrus orchards.

## 2. Materials and Methods

**Figure 1:**
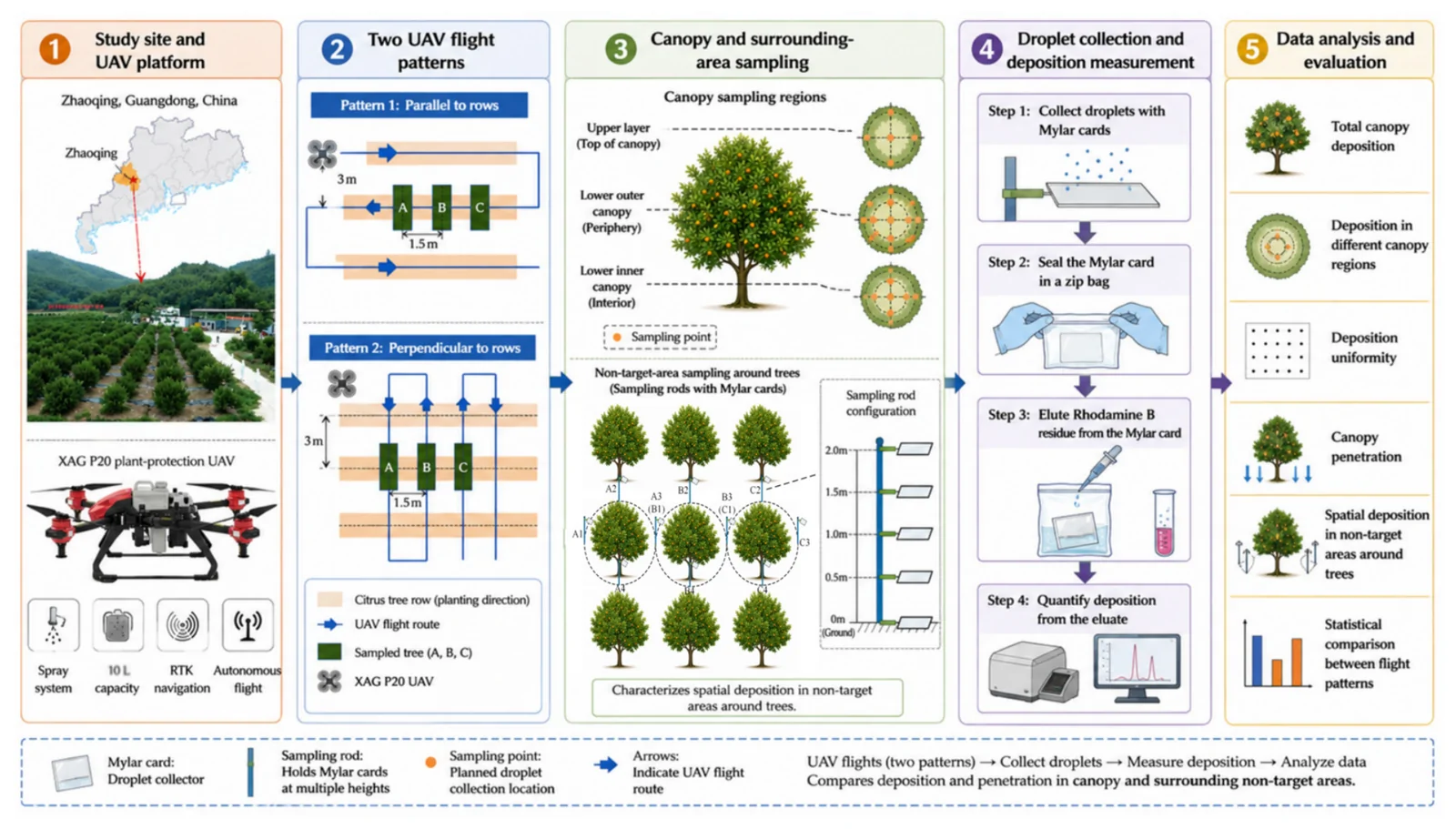
Overall technical workflow of this study.

The overall technical workflow of this study is shown in Figure 1, including the experimental site and equipment, experimental design, sample collection, and droplet deposition measurement. The experimental design was intended to compare droplet deposition characteristics between the two UAV flight patterns under consistent operating conditions.

### 2.1. Experimental Site and Equipment

#### 2.1.1. Experimental Site

**Figure 2:**
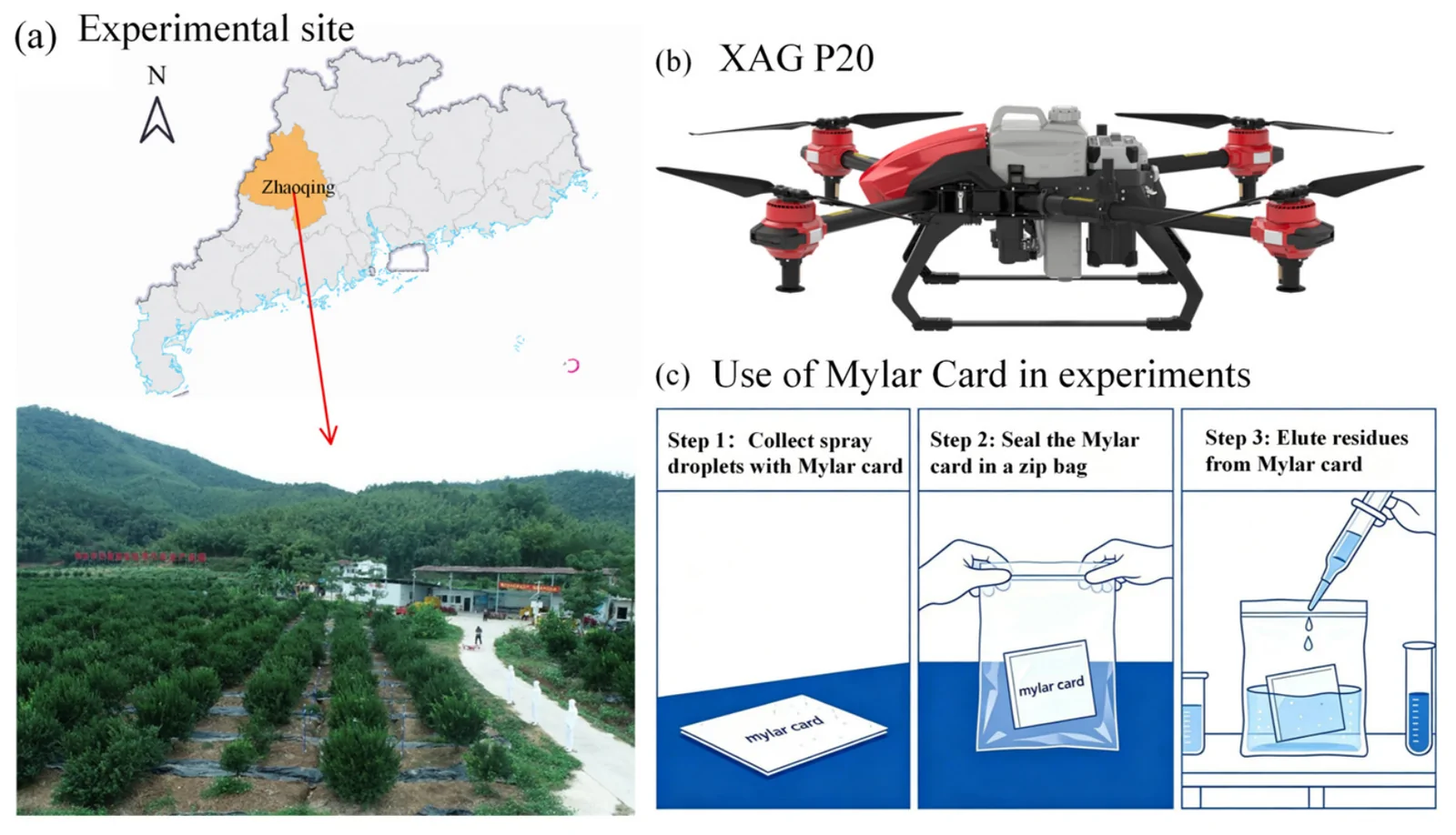
Locations and Equipment. (**a**) Experimental site: Shatangju Provincial Modern Agricultural Industrial Park, Zhaoqing City, Guangdong Province, China. (**b**) XAG P20. (**c**) Use of Mylar Card in experiments.

The experiment was conducted at the Shatangju Provincial Modern Agricultural Industrial Park in Zhaoqing City, Guangdong Province, China (23.4° N, 112.65° E). During the experiment, the ambient temperature was 27.2 °C, the relative humidity was 72.8%, and the wind speed ranged from 0.7 to 1.2 m/s. The spraying experiments for both flight patterns were conducted under comparable meteorological conditions to minimize the influence of environmental variation on the comparison of droplet deposition between treatments. Citrus trees were selected as the experimental subjects, with an average tree height of approximately 2.0–3.0 m, a tree spacing of approximately 1.5 m, and a row spacing of approximately 3.0 m. As shown in Figure 2, the trees in the experimental area were planted in relatively regular rows, and the canopies exhibited a moderate density representative of the structural characteristics of typical citrus orchards in southern China [5].

#### 2.1.2. Experimental Equipment

An XAG P20 UAV (Guangzhou XAG Co., Ltd., Guangzhou, China) was used for the spraying operations, as shown in Figure 2b, and its main technical specifications are listed in Table 1. The P20 is an electric multi-rotor UAV equipped with autonomous flight control and spraying control systems, allowing low-altitude spraying operations to be conducted along preset flight routes. The plant-protection UAV was equipped with four SNZ-14000A centrifugal nozzles, with an atomized droplet-size range of 90–300 μm, an effective spray width of 2–5 m, and a maximum total spray flow rate of 5.6 L/min. Because this UAV uses centrifugal atomization, droplets are generated primarily through the rotation of the atomizing discs; therefore, the spray pressure parameter commonly used for conventional pressure nozzles is not applicable. The same XAG P20 plant-protection UAV was used for both flight patterns, with the nozzle model, number of nozzles, installation positions, spray flow rate, and other major operational parameters kept consistent. Accordingly, the droplet-size characteristics determined by the nozzle atomization system and the rotor-induced airflow conditions had the same equipment basis for both treatments, thereby minimizing the influence of spraying-system differences on the comparison between the two flight patterns.

### 2.2. Experimental Design

#### 2.2.1. Flight Pattern Settings

**Figure 3:**
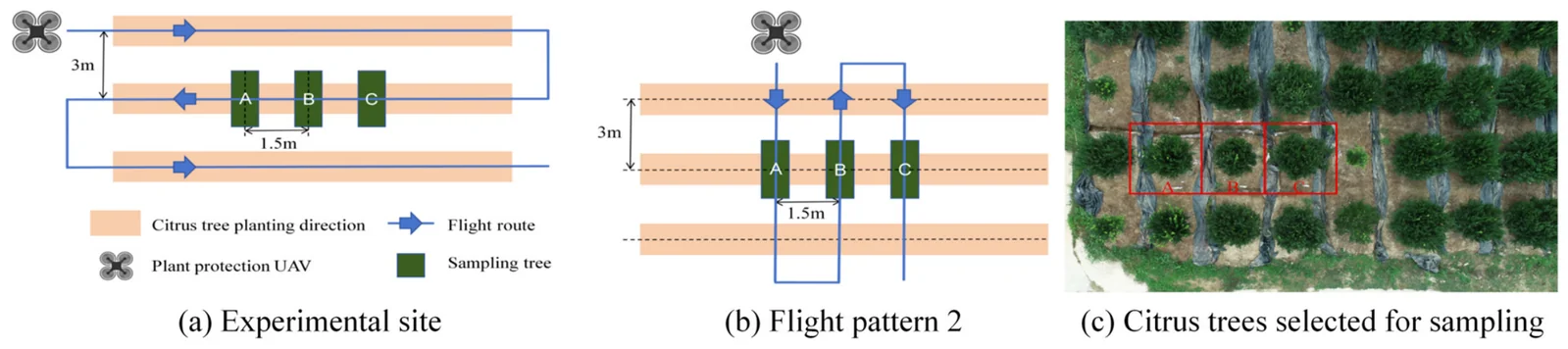
Schematic diagrams of the two flight patterns.

Two flight pattern treatments were designed to compare the effects of changes in the UAV flight direction relative to the citrus tree rows on droplet deposition distribution. In flight pattern 1, the UAV flew parallel to the citrus tree planting direction, passing through the experimental area along the tree rows. In flight pattern 2, the UAV flew perpendicular to the citrus tree planting direction, crossing the tree rows through the experimental area. The two flight patterns are illustrated in Figure 3.

For each flight pattern, three citrus trees with relatively similar growth status and canopy structure were selected as sampling trees and labeled A, B, and C, as shown in Figure 3c. The three citrus trees were treated as tree-level biological replicates, whereas multiple sampling points arranged at different canopy positions within each tree were considered spatial sub samples for characterizing the distribution of droplet deposition within the canopy and were not treated as independent biological replicates. The number of sampled trees, sampling-point arrangement, and sample-labeling scheme were kept consistent between the two flight patterns to ensure comparability between treatments.

To ensure comparability between the two flight pattern treatments, the flight height, flight speed, spray flow rate, and spray solution formulation were kept constant. The flight height was set to 3.0 m above the ground, and the flight speed was set to 1.0 m/s. Based on the flight speed, total spray flow rate, and effective spray width, the application volume used in this experiment was calculated to be 311.1 L/ha. During spraying, the UAV flew at a constant speed along the preset flight route, which was arranged to pass as close as possible to the central area of the target citrus tree canopies to minimize deposition errors caused by flight deviation.

#### 2.2.2. Arrangement of Droplet Sampling Points

To characterize droplet deposition within the citrus canopy and in the surrounding space under different flight patterns, both canopy sampling points and sampling-rod points were arranged. The sampling-point layout, number of sampling points, and naming rules were kept consistent between the two flight pattern treatments.

According to the citrus canopy structure and the requirements for droplet deposition assessment, each citrus canopy was divided into three sampling regions: the upper canopy, the outer lower canopy, and the inner lower canopy. The upper canopy included the canopy top, while the lower canopy was further divided into outer and inner regions to compare the spatial distribution of droplet deposition among different canopy regions. Five sampling points were arranged in the upper layer at the east, west, south, north, and central positions. Four sampling points were arranged in the lower outer layer in the east, west, south, and north directions. Five sampling points were arranged in the lower inner layer at the east, west, south, north, and central positions. Thus, a total of 14 canopy sampling points were arranged on each citrus tree to evaluate the effects of flight pattern on droplet deposition in different canopy layers and on canopy penetration.

**Figure 4:**
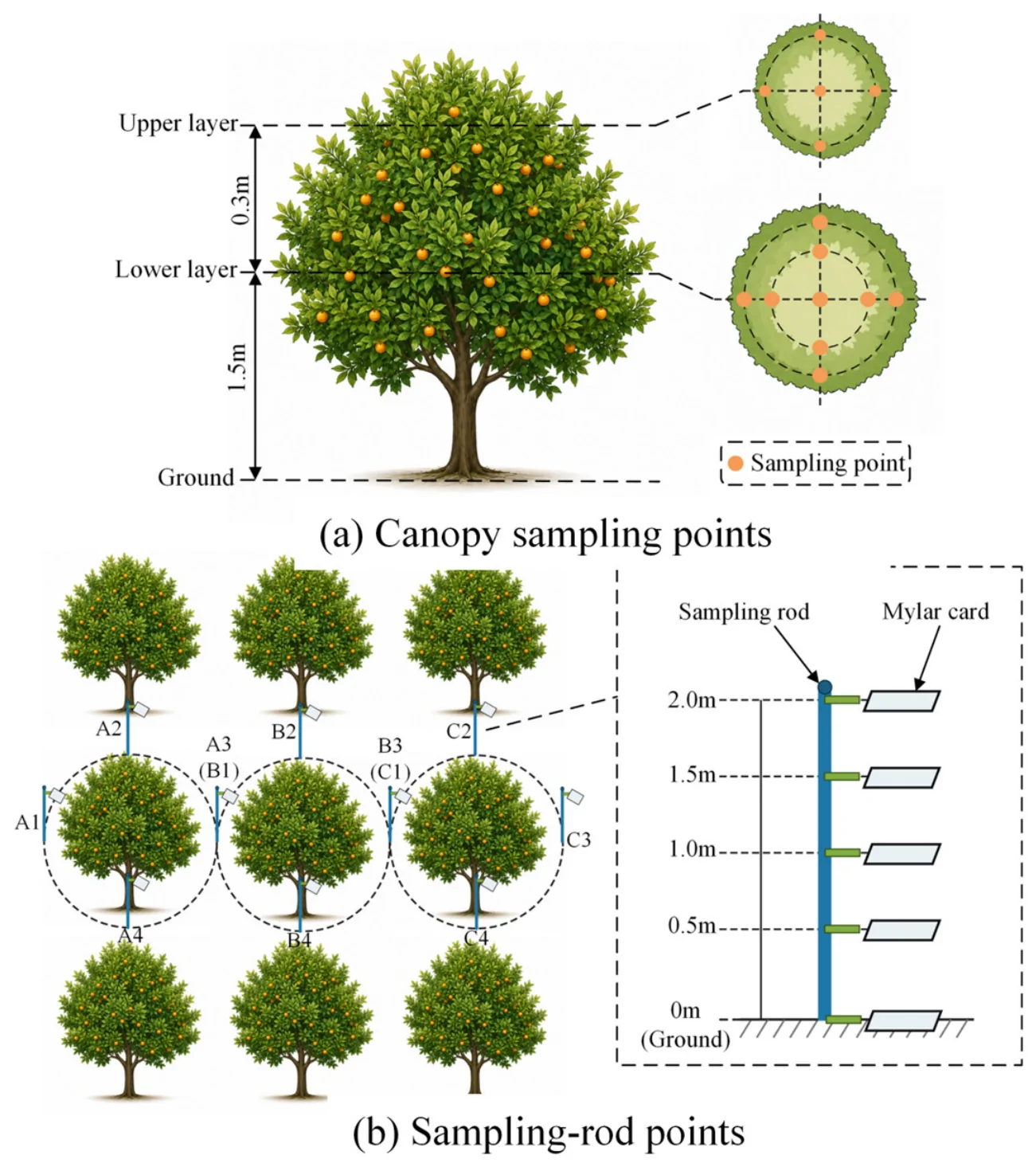
Arrangement of droplet sampling points.

The sampling-rod points were used to evaluate droplet deposition in the space surrounding the trees. Sampling rods were arranged clockwise around the sampled trees, and Mylar cards (Dongguan Jubang Plastic Materials Co., Ltd., Dongguan, China) were fixed to each rod at heights of 0, 0.5, 1.0, 1.5, and 2.0 m above the ground. Sampling points on the rods were used to characterize the spatial distribution of droplet deposition at different heights around the trees under different flight patterns and to compare deposition between locations directly beneath the flight path and those away from the flight path, thereby assessing the spatial concentration of droplet deposition relative to the UAV flight path. The arrangement of the canopy sampling points and sampling-rod points is shown in Figure 4.

### 2.3. Sample Collection and Determination of Deposition Amount

Droplet deposition within the citrus canopies and in the surrounding space was collected using Mylar cards (5 cm × 10 cm). After spraying, the cards were left in place until the deposited droplets had stabilized. The cards were then re-moved sequentially according to their sampling-point codes and placed individually in labeled sealed bags. Each bag was labeled with information including the flight pattern treatment, sampling-tree code, canopy layer, sampling direction, and sampling-rod height to prevent sample confusion. After collection, the samples were immediately stored under low-temperature conditions and transported to the laboratory as soon as possible for further analysis. It should be noted that Mylar cards provide a standardized method for measuring droplet deposition; however, their surface properties differ from those of actual citrus leaves. Therefore, the results obtained in this study are primarily intended for relative comparisons of deposition characteristics between different flight patterns and should not be regarded as equivalent to the actual droplet coverage and retention on leaf surfaces.

Droplet deposition was determined with reference to the spray deposition measurement method specified in ISO 24253-1 [29]. During sample processing, 5 mL of purified water was added to each sealed bag as the eluent. The bags were thoroughly shaken to dissolve the Rhodamine B tracer deposited on the Mylar cards into the eluent. After complete elution, an appropriate volume of the solution was withdrawn using a dis-posable syringe and filtered through a 0.22 μm membrane filter. The filtrate was then transferred to a microplate, and its absorbance was measured at a wavelength of 514 nm using a microplate reader (BioTek Instruments, Inc., Winooski, VT, USA). The Rhodamine B mass concentration in the eluent was calculated from the standard calibration curve according to Equation (1):

(1) $$Y\;=\;0.0283\;X\;+\;0.0043\;(R^{2}\;=\;0.999)$$

where Y is the absorbance measured using the microplate reader; X is the mass concentration of Rhodamine B, μg/mL; and $R^{2}$ is the coefficient of determination of the standard calibration curve.

According to Equation (1), the Rhodamine B mass concentration in the eluent was calculated using Equation (2):

(2) $$Q_{e}\;=\;\frac{\left(\rho_{\mathrm{smpl}}-\rho_{\mathrm{blk}}\right)}{F_{\mathrm{cal}}}$$

where $Q_{e}$ is the Rhodamine B mass concentration in the eluent, μg/mL; $\rho_{\mathrm{smpl}}$ is the absorbance of the sample eluent; $\rho_{\mathrm{blk}}$ is the absorbance of blank distilled water; and $F_{\mathrm{cal}}$ is the slope of the standard calibration curve.

The amount of Rhodamine B deposited on each Mylar card was calculated using Equation (3):

(3) $$M\;={\;Q}_{e}\text{·}V$$

where *M* is the amount of Rhodamine B deposited on a single Mylar card, μg, and *V* is the volume of the eluent, mL.

### 2.4. Data Processing and Evaluation Indices

The experimental data were statistically analyzed using WPS and DPS 7.0 software. The amount of Rhodamine B deposition was used as the indicator of droplet deposition performance. The total canopy deposition, deposition in different canopy layers, canopy deposition uniformity, canopy penetration rate, and rod-position deposition percentage were calculated for the two flight patterns. Differences between treatments were evaluated using one-way analysis of variance, with the significance level set at *p* < 0.05.

The total canopy deposition of an individual citrus tree was used to characterize the overall droplet deposition within the entire canopy and was calculated using Equation (4):

(4) $$M_{t}\;=\sum_{i=1}^{n}M_{i}$$

where $M_{t}$ is the total canopy deposition of an individual citrus tree, μg; $M_{i}$ is the amount of Rhodamine B deposited at the *i* th canopy sampling point, μg; and *n* is the number of canopy sampling points on an individual citrus tree.

Deposition in different canopy layers was used to analyze differences in droplet distribution among the upper layer, lower outer layer, and lower inner layer and was calculated using Equation (5):

(5) $$M_{l}\;=\sum_{j=1}^{n_{l}}M_{j}$$

where $M_{l}$ is the amount of Rhodamine B deposited in a given canopy layer, μg; $M_{j}$ is the amount of Rhodamine B deposited at the *j*-th sampling point in that layer, μg; and $n_{l}$ is the number of sampling points in the corresponding canopy layer.

Canopy deposition uniformity was characterized using the coefficient of variation (*CV*), which was calculated using Equation (6):

(6) $$\mathrm{CV}\;=\frac{\mathrm{SD}}{\bar{M}}\;\times \;100\%$$

where *CV* is the coefficient of variation of deposition, %; *SD* is the standard deviation of deposition under the same treatment, μg; and $\bar{M}$ is the mean deposition under the same treatment, μg. A lower *CV* value indicates a more uniform droplet deposition distribution.

The canopy penetration rate in this study was mainly used to characterize the relative extent of droplet transport from the outer canopy toward the inner region of the lower canopy and was calculated using Equation (7):

(7) $$P_{c}\;=\;\frac{M_{\mathrm{in}}}{M_{t}}\;\times \;100\%$$

where $P_{c}$ is the canopy penetration rate, %; $M_{\mathrm{in}}$ is the deposition in the lower inner layer, μg; and $M_{t}$ is the total canopy deposition of an individual citrus tree, μg. It should be noted that this index is affected by variations in total canopy deposition. Therefore, when evaluating canopy penetration characteristics, the absolute deposition in the inner canopy was also considered for a comprehensive assessment.

The rod-position deposition percentage was used to characterize the proportion of deposition at each sampling height relative to the total deposition on the same sampling rod and was calculated using Equation (8):

(8) $$P_{h}\;=\;\frac{M_{h}}{M_{p}}\;\times \;100\%$$

where $P_{h}$ is the deposition percentage at a given sampling height, %; $M_{h}$ is the amount of Rhodamine B deposited at that height, μg; and $M_{p}$ is the total deposition measured at all sampling heights on the corresponding sampling rod, μg.

## 3. Results and Analysis

### 3.1. Droplet Deposition in Citrus Canopies Under Different Flight Patterns

**Figure 5:**
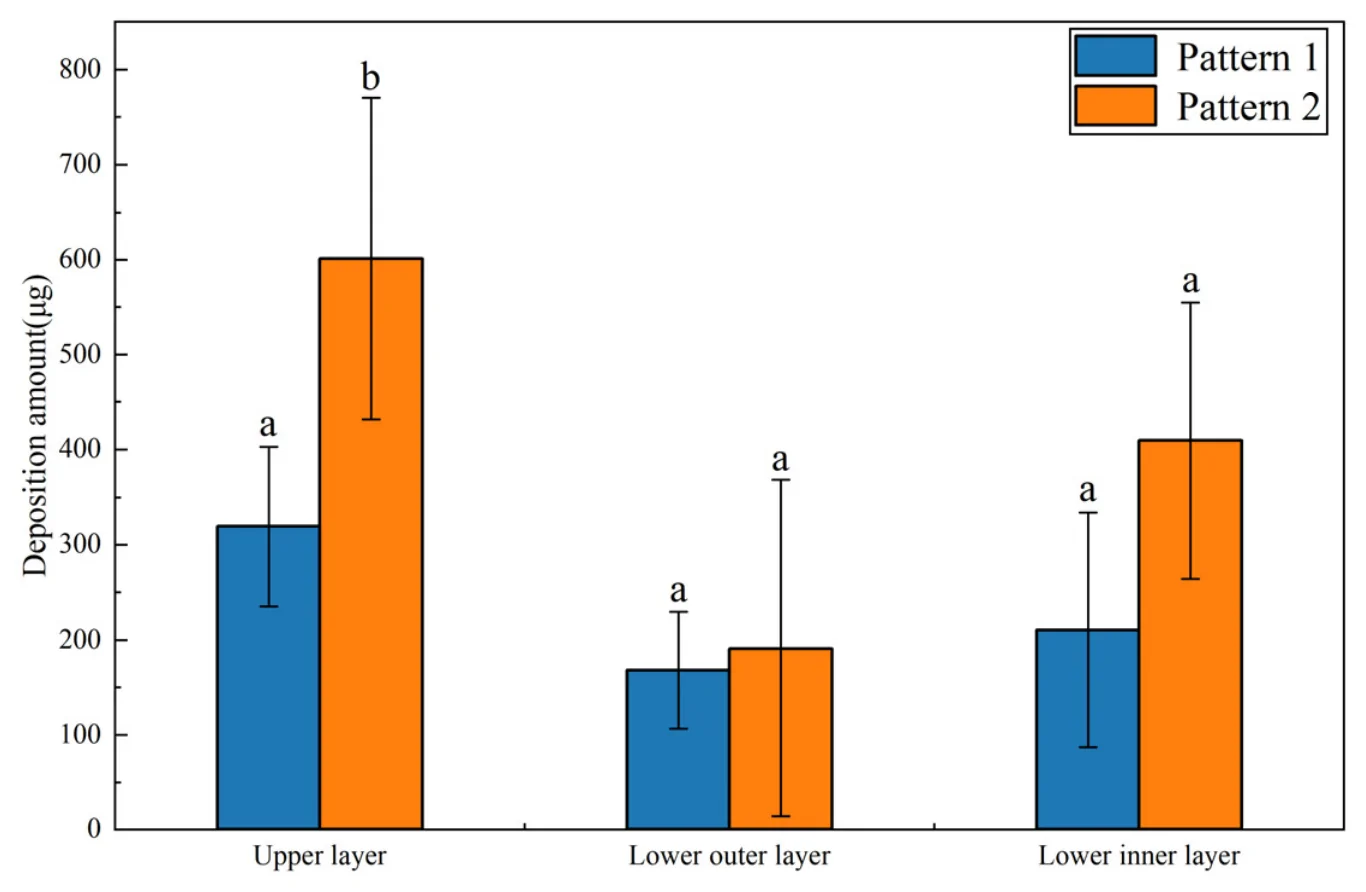
Total droplet deposition in different canopy layers under the two flight patterns. Different lowercase letters indicate significant differences between the two flight patterns within the same canopy layer (*p* < 0.05).

Droplet deposition in different canopy layers under the two flight patterns is presented in Table 2 and Figure 5. Under flight pattern 1, the mean deposition amounts in the upper layer, lower outer layer, and lower inner layer were 318.85, 168.01, and 210.64 μg, respectively, with corresponding standard deviations of 83.67, 61.70, and 123.50 μg. Under flight pattern 2, the mean deposition amounts in the upper layer, lower outer layer, and lower inner layer were 600.74, 190.98, and 409.30 μg, respectively, with corresponding standard deviations of 169.16, 177.33, and 145.34 μg. Compared with flight pattern 1, flight pattern 2 increased deposition in the upper layer and lower inner layer by 88.40% and 94.31%, respectively, whereas deposition in the lower outer layer increased by only 13.67%.

As shown in Figure 5, the upper layer exhibited the highest droplet deposition under both flight patterns, indicating that the upper canopy was more likely to directly intercept spray droplets during low-altitude UAV spraying. Using the individual citrus tree as the replicate (*n* = 3), sampling points within the same tree were treated as spatial subsamples. Deposition in different canopy regions was first calculated for each individual tree, and differences between the two flight patterns were then compared using tree-level data, paired-samples t-tests were conducted to compare deposition between the two flight patterns in each canopy layer. In the upper layer, deposition under flight pattern 2 (600.74 ± 169.16 μg) was significantly higher than that under flight pattern 1 (318.85 ± 83.67 μg), with a statistically significant difference (*t* = −5.35, *df* = 2, *p* = 0.033). In the lower outer layer, deposition under flight patterns 1 and 2 was 168.01 ± 61.70 μg and 190.98 ± 177.33 μg, respectively, with no significant difference (*p* = 0.806). Similarly, deposition in the lower inner layer was 210.64 ± 123.50 μg under flight pattern 1 and 409.30 ± 145.34 μg under flight pattern 2, with no significant difference (*p* = 0.329). Nevertheless, the markedly higher deposition in the lower inner layer under flight pattern 2 indicates that flying perpendicular to the citrus tree planting direction facilitated droplet entry into the inner canopy and improved internal canopy deposition.

The total canopy deposition of citrus trees under the two flight patterns is shown in Table 3. The mean total canopy deposition under flight patterns 1 and 2 was 697.49 and 1201.01 μg, respectively, with corresponding standard deviations of 159.19 and 36.86 μg. Compared with flight pattern 1, flight pattern 2 increased total canopy deposition by 72.19%. The smaller variation among the three sampled trees under flight pattern 2 also indicated greater stability in total canopy deposition.

One-way analysis of variance and Duncan’s multiple range test showed that flight pattern had a significant effect on total canopy deposition (*F* = 28.486, *p* = 0.0059). Total canopy deposition under flight pattern 2 was significantly higher than that under flight pattern 1, indicating that, under the present experimental conditions, flying perpendicular to the citrus tree planting direction was more conducive to increasing overall droplet deposition within the canopy.

### 3.2. Uniformity of Canopy Deposition Under Different Flight Patterns

**Figure 6:**
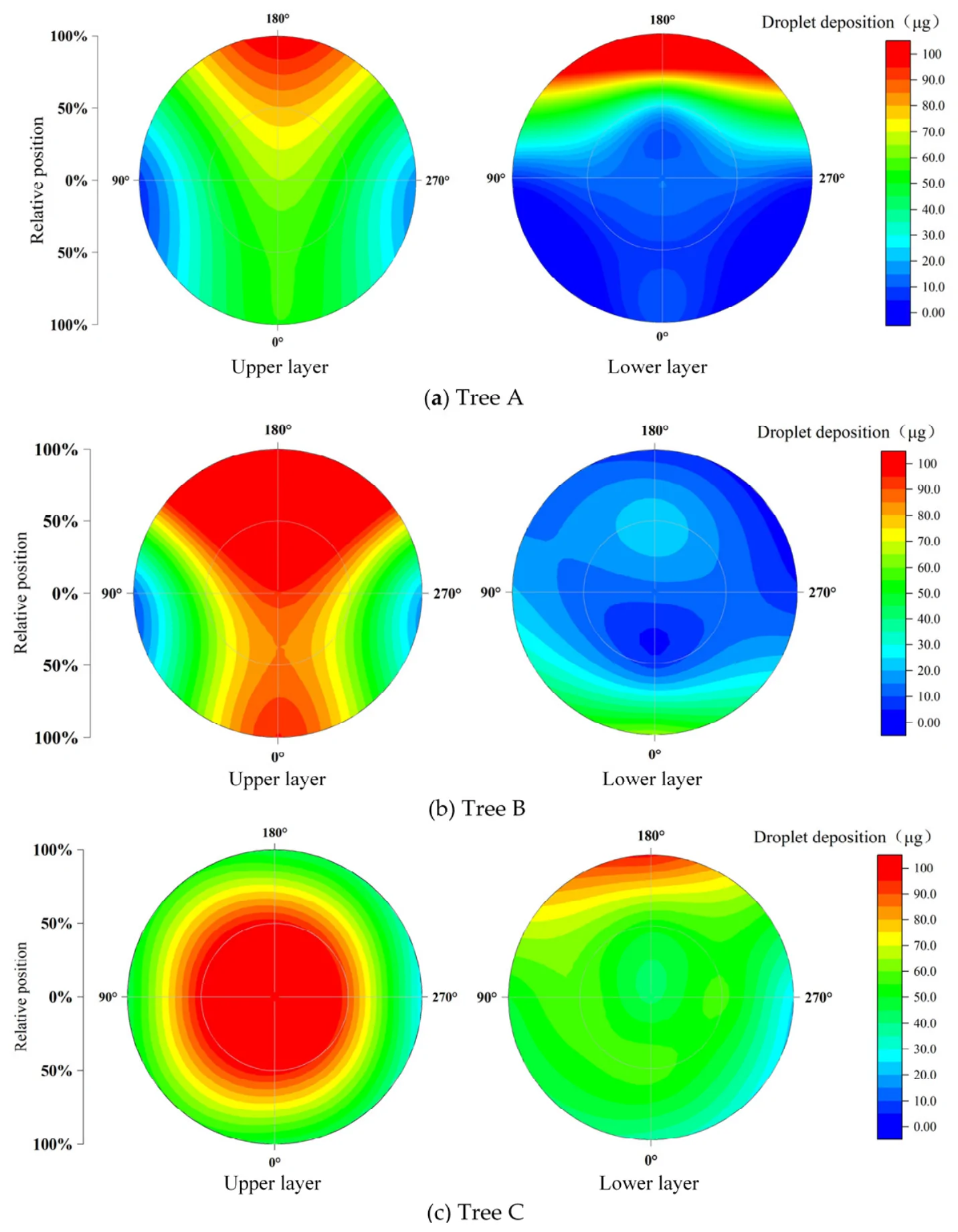
Radar contour maps of droplet deposition in different canopy layers under flight pattern 1.

To compare the effects of the two flight patterns on droplet deposition distribution within the citrus canopy, radar contour maps of droplet deposition were generated for the three sampled trees (Trees A, B, and C) under flight pattern 1, in which the UAV flew parallel to the tree-row direction, and flight pattern 2, in which the UAV flew perpendicular to the tree-row direction, as shown in Figure 5 and Figure 6. In the radar contour maps, the radial axis represents deposition amount, whereas the angular axis represents sampling direction and corresponds to different canopy orientations.

As shown in Figure 6, under flight pattern 1, all three citrus trees exhibited substantially greater droplet deposition in the upper layer than in the lower layer in the vertical direction, indicating that droplets were mainly deposited in the upper canopy and that canopy penetration was limited. In the horizontal direction, pronounced directional differences were observed among the three trees. Droplet deposition in the 0° and 180° directions was greater than that in the 90° and 270° directions, indicating an evident directional deposition bias under flight pattern 1.

**Figure 7:**
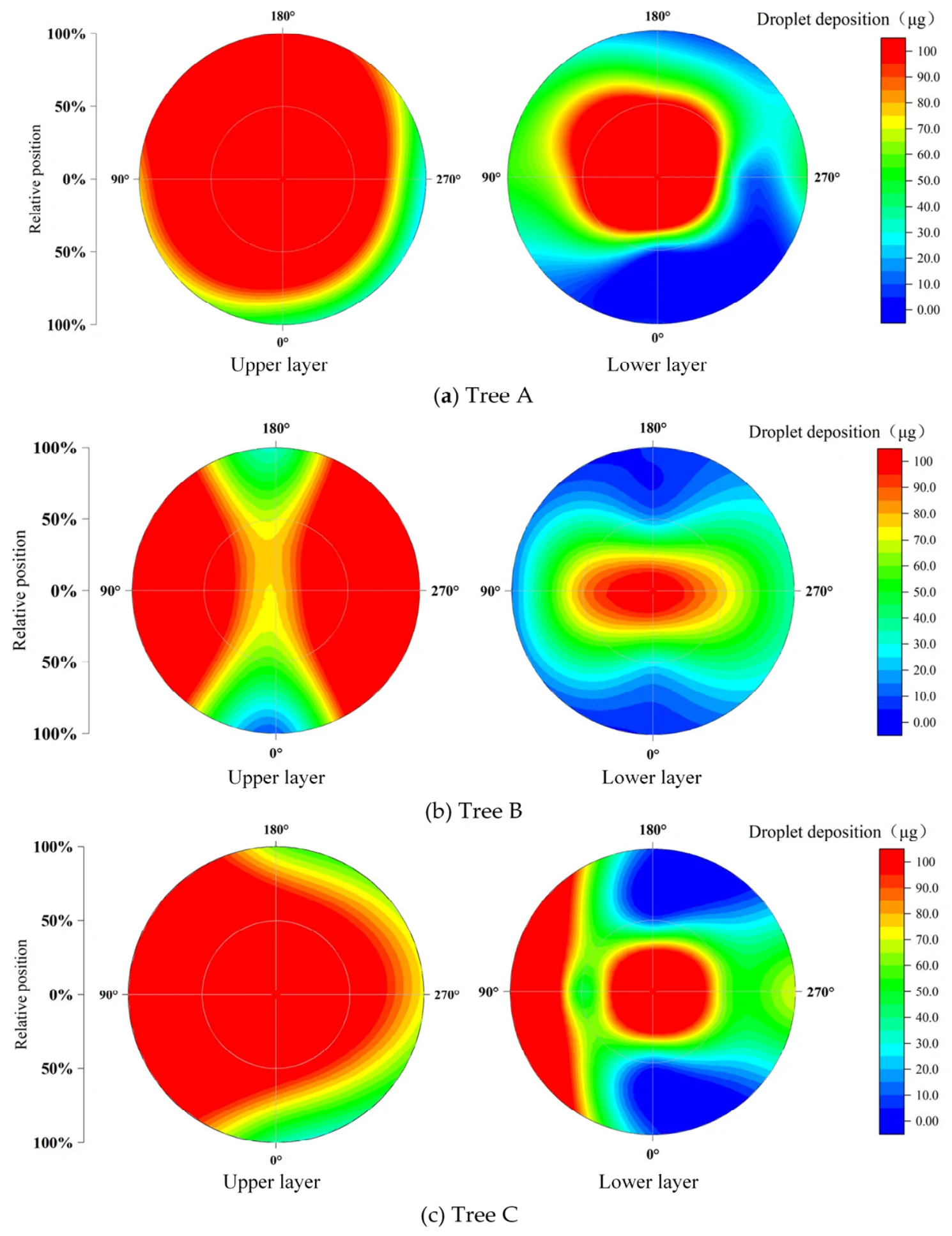
Radar contour maps of droplet deposition in different canopy layers under flight pattern 2.

As shown in Figure 7, under flight pattern 2, the difference in droplet deposition between the upper and lower layers of the three citrus trees was markedly smaller than that under flight pattern 1. This result indicates that droplets more effectively reached the lower canopy under flight pattern 2, thereby improving canopy penetration. In the horizontal direction, droplet deposition under flight pattern 2 also exhibited a certain degree of directional variation. Deposition in the 90° and 270° directions was greater than that in the 0° and 180° directions. However, the directional deposition bias was weaker than that observed under flight pattern 1, and deposition in the central region of the lower layer was increased relative to that under flight pattern 1.

**Figure 8:**
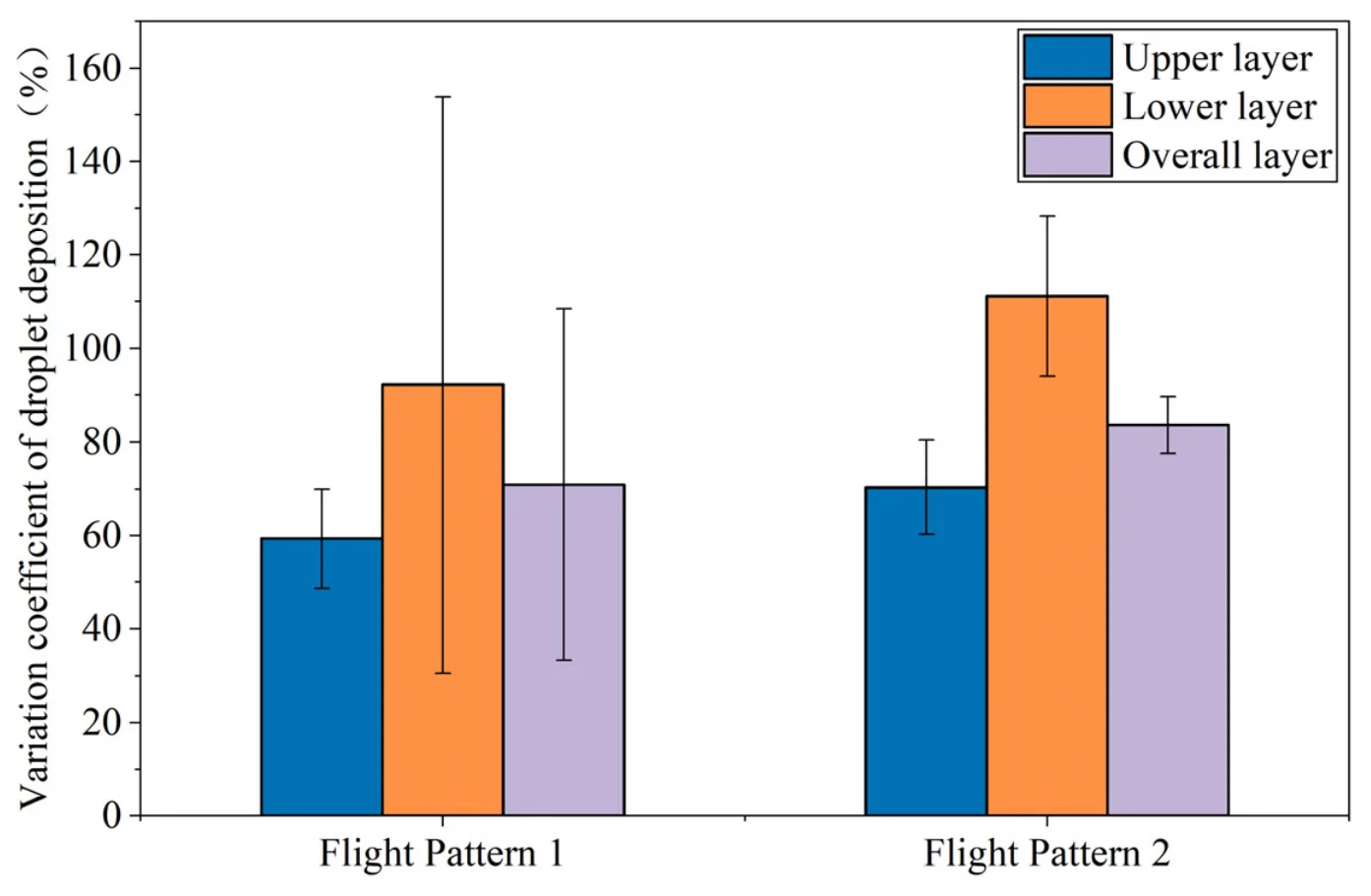
Coefficients of variation of droplet deposition in different canopy layers under the two flight patterns.

The coefficient of variation (*CV*) of droplet deposition is an important indicator of spray uniformity, with a lower *CV* indicating a more uniform droplet distribution. Figure 8 presents the *CV* values of droplet deposition in the upper layer, lower layer, and overall canopy under the two flight patterns.

Under flight pattern 1, the *CV* of droplet deposition in the lower layer was markedly higher than that in the upper layer. The *CV* values were 59.32% for the upper layer, 92.20% for the lower layer, and 70.91% for the overall canopy. These results indicate relatively poor deposition uniformity in the lower layer, with pronounced spatial variation in droplet deposition. Under flight pattern 2, the *CV* values were 70.34% for the upper layer, 111.13% for the lower layer, and 85.52% for the overall layer. Similar to flight pattern 1, the lower layer exhibited a higher *CV* than the upper layer, and the degree of variation was more pronounced.

In addition, the *CV* of the overall layer under flight pattern 2 was greater than that under flight pattern 1, indicating that droplet deposition uniformity decreased under flight pattern 2. An independent-samples *t*-test showed no significant difference in overall-canopy *CV* between the two flight patterns (*t* = −0.42, *df* = 2, *p* = 0.712). These results indicate that, under the present experimental conditions, changing the flight pattern did not significantly affect the uniformity of droplet deposition within the citrus canopy.

### 3.3. Canopy Penetration Under Different Flight Patterns

Canopy penetration rate is a key indicator for evaluating the ability of droplets to enter the inner canopy. A higher value indicates that droplets are more likely to reach the lower and inner canopy regions. The calculated canopy penetration rates under the two flight patterns are presented in Table 4.

As shown in Table 4, the mean canopy penetration rates under flight patterns 1 and 2 were 28.23% and 34.15%, respectively. Meanwhile, the mean deposition in the inner region of the middle-to-lower canopy under Pattern 2 was 409.30 μg, which was higher than the 210.64 μg observed under Pattern 1. Therefore, Pattern 2 exhibited not only a higher canopy penetration rate but also a markedly higher absolute deposition level within the inner canopy. Compared with flight pattern 1, flight pattern 2 increased the canopy penetration rate by 20.97%, indicating that when the UAV flew perpendicular to the citrus tree planting direction, a greater proportion of droplets entered the lower inner layer of the canopy.

For the individual trees, the canopy penetration rates of Trees A, B, and C under flight pattern 1 were 13.37%, 36.68%, and 34.66%, respectively, whereas those under flight pattern 2 were 48.51%, 26.91%, and 27.04%, respectively. Differences in canopy penetration rate were observed among the three sampled trees, indicating that canopy penetration was influenced not only by the flight pattern but also potentially by individual variations in canopy morphology and the spatial distribution of branches and leaves. The quantitative relationships between these canopy characteristics and droplet penetration require further investigation by incorporating structural parameters such as canopy density and leaf area. Overall, flight pattern 2 increased total canopy deposition and, to some extent, improved droplet penetration into the inner canopy.

### 3.4. Distribution Characteristics of Rod-Position Deposition Under Different Flight Patterns

**Figure 9:**
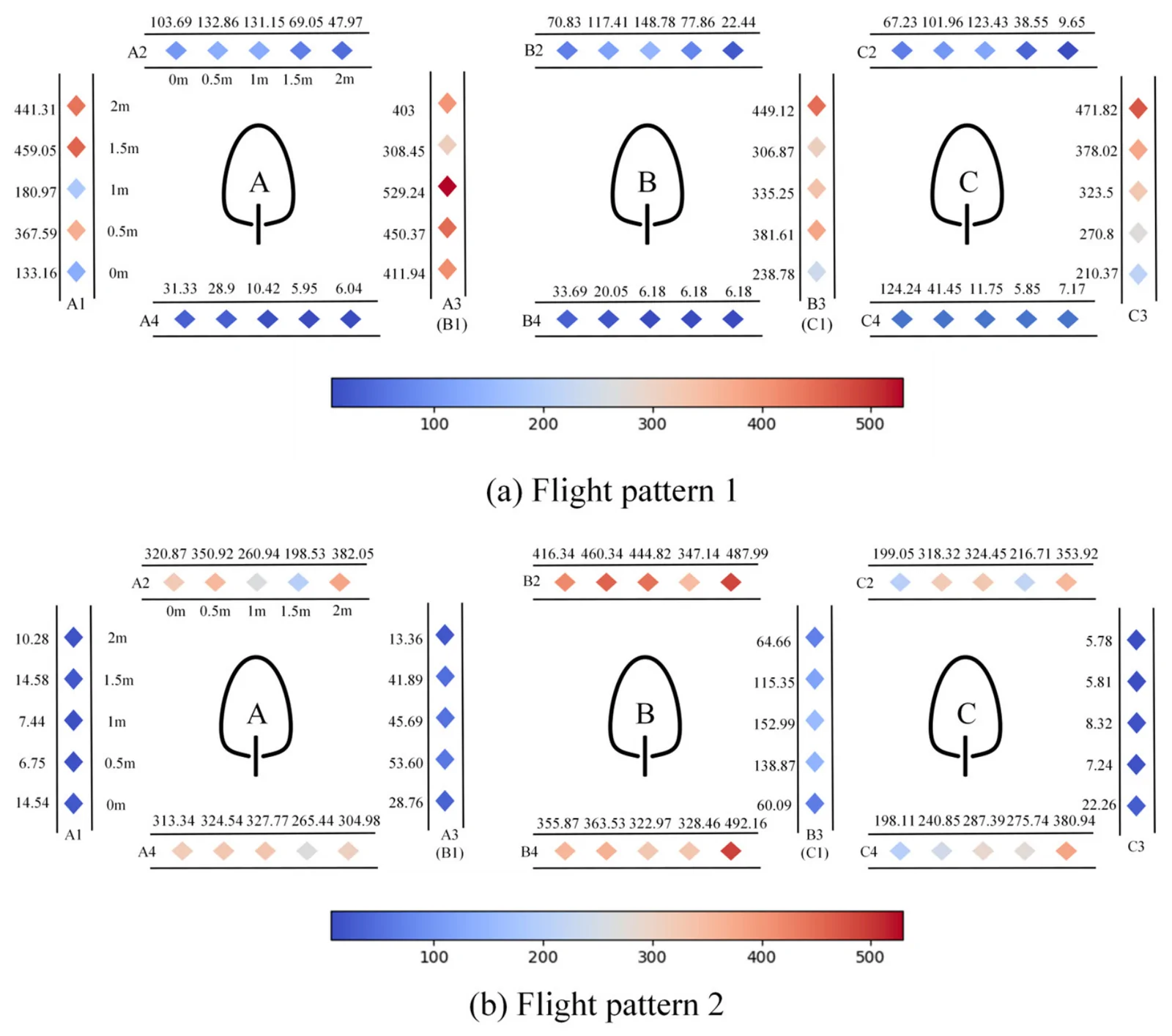
Distribution of droplet deposition at different rod heights under the two flight patterns.

**Figure 10:**
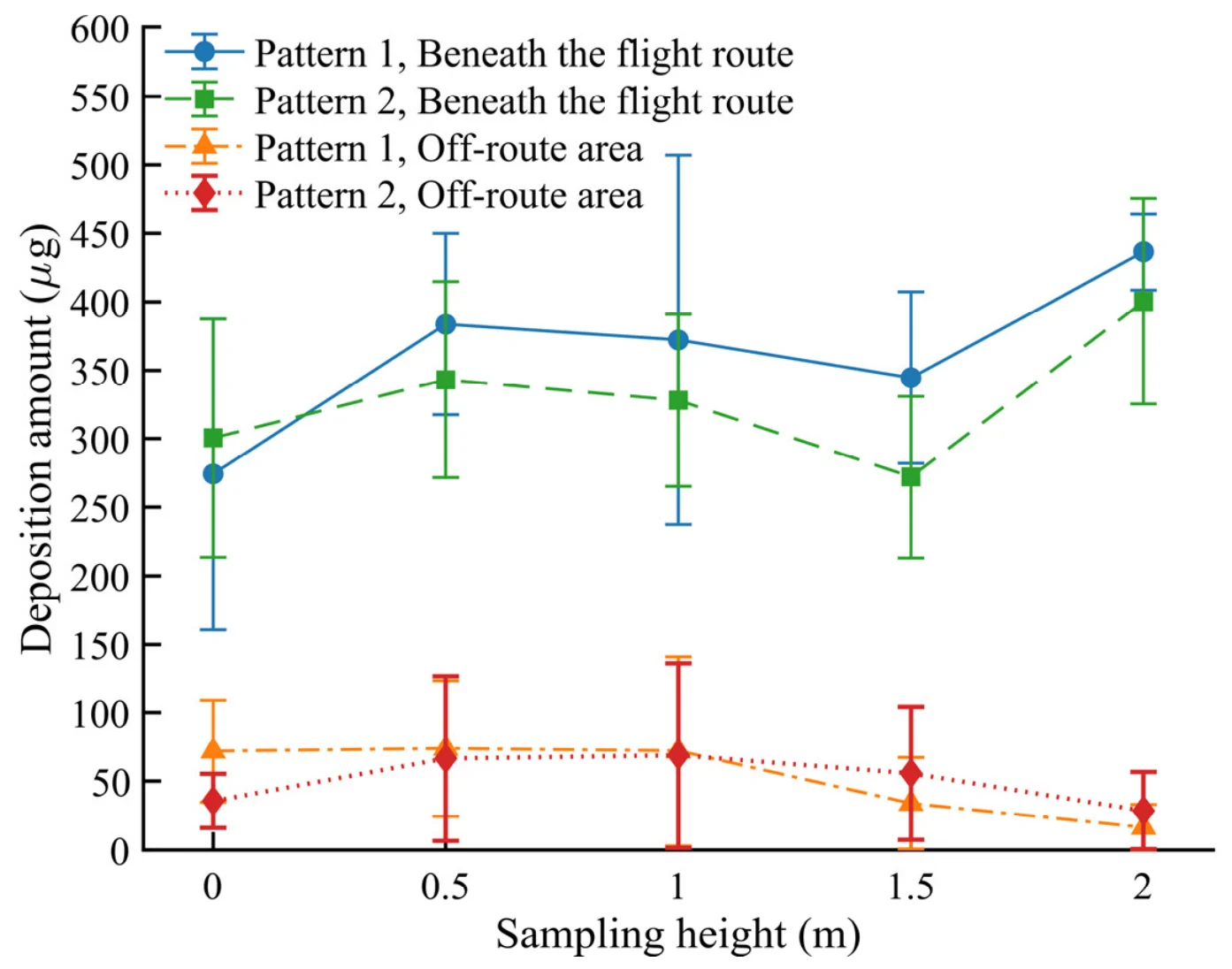
Droplet deposition at different sampling heights on the sampling rods under the two flight patterns.

The rod-position deposition amounts and the corresponding significance analysis under the two flight patterns are presented in Table 5. The spatial distribution of droplet deposition at different rod heights is shown in Figure 9, and the variation in deposition amount with sampling height is shown in Figure 10. As indicated in Table 5, rod-position deposition under both flight patterns exhibited a clear dependence on the flight route. Droplets were mainly deposited beneath the flight route, whereas deposition in the off-route area was relatively low.

Under flight pattern 1, the mean rod-position deposition amount beneath the flight route was 1810.97 μg, while that in the off-route area was 268.01 μg. Under flight pattern 2, the corresponding mean deposition amounts were 1644.08 μg and 255.59 μg, respectively. Thus, the deposition amount beneath the flight route was approximately 6.8 times that in the off-route area under flight pattern 1 and 6.4 times that under flight pattern 2.

One-way analysis of variance showed that deposition differed significantly among rod-position areas (*F* = 76.235, *p* = 0.0001). Duncan’s multiple range test further indicated that, under both flight patterns, deposition beneath the flight route was significantly greater than that in the off-route area. This result demonstrates that droplets were mainly concentrated near the flight route during UAV spraying, and that the flight route directly affected the distribution of high-deposition zones in the space surrounding the trees.

In terms of deposition proportion, the area beneath the flight route accounted for 87.11% and 86.55% of the total rod-position deposition under flight patterns 1 and 2, respectively, whereas the off-route area accounted for only 12.89% and 13.45%. These results further confirm the pronounced route-dependent concentration of droplet deposition.

Rod-position deposition fluctuated with sampling height and did not exhibit a monotonic increasing or decreasing trend. The deposition amount at each height in Figure 9 was calculated from the sampling points within the corresponding rod-position area, with shared rods classified separately according to the adjacent sampled trees. As shown in Figure 9 and Figure 10, local high and low deposition values occurred at different heights under both flight patterns, indicating that the vertical distribution of droplet deposition was jointly affected by the location of the flight route, downwash airflow disturbance, and the local airflow environment. Overall, the rod-position results show that droplets did not disperse uniformly around the trees, but instead exhibited a pronounced concentration near the flight route. This finding also helps explain the differences in total canopy deposition and inner-canopy deposition observed between the two flight patterns.

## 4. Discussion

The results of this study showed that flight pattern significantly affected droplet deposition within citrus canopies. Compared with flight pattern 1, total canopy deposition under flight pattern 2 increased from 697.49 μg to 1201.01 μg, representing an increase of 72.19%. Meanwhile, deposition in the upper layer and lower inner layer under flight pattern 2 increased by 88.40% and 94.31%, respectively, compared with flight pattern 1. These results indicate that when the UAV flew perpendicular to the citrus tree planting direction, it not only increased overall canopy deposition but also enhanced droplet deposition within the inner canopy. Therefore, in citrus orchard spraying operations, flight pattern is not merely a route-planning issue, but also an important factor affecting droplet deposition efficiency and canopy penetration [8,19]. In addition, it should be noted that an increase in canopy deposition does not necessarily correspond to a proportional improvement in pesticide use efficiency, which is also influenced by factors such as application volume per unit area, deposition uniformity, non-target deposition, and pest-control efficacy. Therefore, the present results should be interpreted as relative differences in droplet deposition between the two UAV flight patterns rather than as evidence of differences in overall plant-protection efficiency. In this study, the same UAV platform and spraying parameters were used for both flight patterns; therefore, the higher canopy deposition observed under Pattern 2 primarily indicates a greater level of droplet deposition within the canopy under the experimental conditions. The actual spraying performance should still be evaluated comprehensively by considering deposition uniformity and the spatial distribution of non-target deposition.

Differences in droplet deposition between the two flight patterns may be related to the position of the flight path and the spatial coverage of the spray. Under Pattern 1, in which the UAV flew parallel to the citrus tree rows, droplets were mainly distributed along the row direction. Under Pattern 2, in which t16he UAV flew perpendicular to the tree rows, the flight path successively crossed the tree canopies and inter-row spaces, thereby altering the flight-route orientation and spatial coverage of the spray and potentially affecting droplet distribution in the upper canopy, inner canopy regions, and surrounding areas. Considering the higher total canopy deposition and greater deposition in the inner region of the middle-to-lower canopy under Pattern 2, the results indicate that different flight patterns can significantly alter the spatial distribution of droplets within different canopy regions.

In addition, different flight directions may affect droplet deposition by altering the interaction between rotor-induced downwash airflow and the tree-row structure. When the UAV flies along the tree rows, it continuously acts on a relatively continuous canopy belt, and the downwash airflow, after being obstructed by the canopy, may spread along the row direction and toward both sides of the canopy. In contrast, when the UAV flies perpendicular to the tree rows, it successively crosses the canopy and inter-row gaps, resulting in alternating spatial boundaries for the downwash airflow, which may further influence airflow transport into the inner canopy and inter-row regions. Therefore, the coupled interaction between rotor-induced airflow and canopy structure under different flight directions may further affect droplet transport and deposition within different canopy regions and in the space surrounding the trees.

Previous studies have also shown that droplet deposition responses vary among different orchard types and UAV systems, primarily due to differences in canopy morphology, foliage density, tree and row spacing, rotor configuration, nozzle arrangement, and operational parameters. These factors jointly influence downwash airflow, droplet transport, and canopy interception processes, thereby resulting in different canopy deposition patterns [30,31]. In the present study, the higher deposition observed in the upper canopy and inner region of the lower canopy under Pattern 2 further suggests that the spatial relationship between the flight pattern and canopy geometry can influence the ability of droplets to penetrate into the canopy.

The canopy penetration results further showed that the mean canopy penetration rate under flight pattern 2 was 34.15%, which was higher than the 28.23% observed under flight pattern 1. This indicates that flying perpendicular to the citrus tree planting direction improved, to some extent, the ability of droplets to reach the lower inner layer. However, the increase in canopy penetration was smaller than the increase in total canopy deposition, suggesting that a higher total deposition does not necessarily result in a more uniform distribution among canopy layers. The coefficient of variation of total canopy deposition among the three sampled trees was lower under flight pattern 2, indicating better overall deposition stability. Nevertheless, the relatively high coefficient of variation in the lower outer layer suggests that considerable spatial variability remained at local canopy positions. Therefore, the performance of UAV spraying in citrus orchards should not be evaluated solely on the basis of total canopy deposition, but should also consider deposition in different canopy layers, canopy penetration, rod-position deposition, and deposition uniformity [32].

The rod-position deposition results further confirmed the route-dependent concentration of droplet deposition. Under both flight patterns, deposition beneath the flight route was significantly greater than that in the off-route area. The deposition beneath the flight route was approximately 6.8 and 6.4 times that in the off-route area under flight patterns 1 and 2, respectively, and accounted for 87.11% and 86.55% of total rod-position deposition. These results indicate that the position of the flight path has a pronounced influence on the formation of high-deposition zones around the trees. Considering the differences in flight-path orientation and spatial arrangement between the two flight patterns, the higher canopy deposition observed under Pattern 2 may result from the combined effects of flight direction, route configuration, and spatial spray coverage. In addition, the smaller flight-path spacing under the perpendicular flight pattern may increase local spray overlap and repeated canopy exposure, thereby contributing to increased canopy deposition. At different sampling heights, deposition on the sampling rods showed some vertical variability, without a monotonic increase or decrease with height. Such vertical fluctuations in deposition may also be associated with local disturbances of rotor-induced downwash airflow within the canopy and inter-row spaces. Previous studies have shown that rotor-induced airflow can alter droplet velocity, dispersion pathways, and deposition locations. Therefore, the interaction between airflow and tree-row structure under different flight directions may be one of the important factors contributing to the observed differences in local droplet deposition patterns [33]. Therefore, rod-position deposition reflects not only the control exerted by the flight route over the droplet deposition zone, but also helps explain the differences in total canopy deposition and inner-canopy deposition between the two flight patterns. The proportions of rod-position deposition recorded in the off-route area were 12.89% and 13.45% under flight patterns 1 and 2, respectively, indicating that the relative lateral dispersion of droplets beyond the directly treated flight routes was similar under the tested conditions. However, the sampling rods were not specifically arranged to determine the effective spray-swath width or spray-swath overlap. Therefore, these results should not be interpreted as evidence that the two flight patterns had equivalent degrees of spray overlap. The closer flight-path spacing used in the perpendicular pattern, together with the resulting potential for spray-swath overlap and repeated canopy exposure, may still have contributed to the higher canopy deposition observed under this pattern.

This study has several limitations. First, only two complete operational flight patterns were compared. Because flight direction, flight-path spacing, and potential spray-swath overlap were not controlled as independent factors, the observed differences in deposition cannot be attributed exclusively to flight direction. In particular, the closer flight-path spacing used in the perpendicular flight pattern may have resulted in greater spray-swath overlap or repeated exposure of some canopy areas. Therefore, the present results reflect the combined effects of flight direction and spatial coverage geometry under the tested operational conditions. Second, three citrus trees were selected as tree-level replicates for each flight pattern, which enabled comparison of droplet deposition characteristics between the different flight patterns under the experimental conditions. However, given the inherent variability in canopy structure among orchard trees, the relatively small sample size may limit the representativeness of the results at a larger orchard scale. Future studies should increase the number of sampled trees and expand the range of replicates across different spatial locations and canopy structures, while independently controlling flight direction, flight-path spacing, effective spray width, application volume per unit area, and the actual spray exposure of the canopy, so as to verify the stability of droplet deposition patterns under different flight modes and clarify the independent effects of these factors on canopy droplet deposition. Furthermore, this study compared only two typical flight patterns, namely, flights parallel and perpendicular to the tree rows. In practical operations, however, the selection of an appropriate flight strategy may also be jointly influenced by orchard geometric and operational factors, such as canopy size, tree and row spacing, flight-path spacing, and spray-swath overlap. Future studies should further investigate the relationship between flight patterns and droplet deposition under different orchard structures by incorporating oblique flight, multiple-pass spraying, and different degrees of spray-swath overlap. In addition, computational fluid dynamics (CFD) simulations or airflow measurements can be integrated with field spraying experiments and compared with the classic sprayer to further analyze rotor-induced airflow, droplet transport, and canopy interception processes, thereby deepening the mechanistic understanding of how different flight patterns affect droplet deposition.

## 5. Conclusions

This study compared the droplet deposition characteristics under two flight patterns: the UAV flying parallel to the citrus tree planting direction and flying perpendicular to the citrus tree planting direction. The effects of flight pattern on total canopy deposition, deposition in different canopy layers, canopy deposition uniformity, canopy penetration, and rod-position deposition distribution were analyzed. The results showed that, under the UAV configuration and citrus orchard planting conditions used in this study, flight pattern had a significant effect on droplet deposition within the citrus canopy. In particular, Pattern 2, in which the UAV flew perpendicular to the citrus tree rows, was more favorable for increasing total canopy deposition and deposition within the inner canopy. It should be emphasized that the conclusions of this study only reflect the relative effects of the two UAV flight patterns on droplet deposition under the experimental conditions and should not be interpreted as an evaluation of overall plant-protection efficiency.

The total canopy deposition under flight patterns 1 and 2 was 697.49 and 1201.01 μg, respectively. Compared with flight pattern 1, flight pattern 2 increased total canopy deposition by 72.19%, and the difference between the two flight patterns was significant (*F* = 28.486, *p* = 0.0059). Deposition in the upper layer, lower outer layer, and lower inner layer under flight pattern 2 was greater than that under flight pattern 1. In particular, deposition in the upper layer and lower inner layer increased by 88.40% and 94.31%, respectively. The mean canopy penetration rate under flight pattern 2 was 34.15%, which was higher than the 28.23% observed under flight pattern 1, indicating that flying perpendicular to the citrus tree planting direction increased the proportion of droplets entering the inner canopy. The rod-position deposition results showed that droplet deposition under both flight patterns exhibited a pronounced concentration near the flight route. Deposition beneath the flight route was approximately 6.8 and 6.4 times that in the off-route area under flight patterns 1 and 2, respectively, and accounted for 87.11% and 86.55% of total rod-position deposition. These results indicate that droplets were mainly deposited near the flight route.

Overall, under the present experimental conditions, flying perpendicular to the citrus tree planting direction effectively increased total canopy deposition and inner-canopy deposition and improved canopy penetration to some extent. However, this study still has certain limitations. Only two typical flight patterns were compared, and the sample size and range of flight-route configurations were relatively limited. Factors such as canopy structure and flight-path spacing were not independently quantified, and no systematic sampling covering all inter-tree and inter-row areas or comparison with a conventional orchard sprayer was conducted. Future studies should expand the sample size, incorporate more practical flight routes, and combine canopy structural parameters, airflow measurements, and CFD simulations to further improve the representativeness of the results and deepen the understanding of droplet transport and deposition mechanisms in plant-protection UAV spraying.

## Figures and Tables

**Table 1:** Main technical specifications of the XAG P20.

Major Parameters	Specification Index
Dimension	1830 × 1822 × 452 mm
Number of rotors	4
Medicine box payload	10 L
Overall weight (unloaded)	13.1 kg
Maximum flow rate	5.6 L/min
Hover accuracy (good GNSS signal)	Horizontal ± 10 cm, Vertical ± 10 cm

**Table 2:** Droplet deposition in different canopy layers under the two flight patterns.

Flight Pattern	Canopy Layer	Tree A Deposition/μg	Tree B Deposition/μg	Tree C Deposition/μg	Mean/μg
Flight pattern 1	Upper layer	246.13	410.30	300.12	318.85 ± 83.67
	Lower outer layer	199.33	96.93	207.76	168.01 ± 61.70
	Lower inner layer	68.73	293.83	269.35	210.64 ± 123.50
Flight pattern 2	Upper layer	492.97	795.71	513.55	600.74 ± 169.16
	Lower outer layer	119.19	60.80	392.95	190.98 ± 177.33
	Lower inner layer	576.70	315.27	335.92	409.30 ± 145.34

**Table 3:** Total canopy deposition of citrus trees under the two flight patterns.

Flight Pattern	Tree A Deposition/μg	Tree B Deposition/μg	Tree C Deposition/μg	Mean/μg
Flight pattern 1	514.19	801.06	777.23	697.49 ± 159.19 a
Flight pattern 2	1188.85	1171.77	1242.42	1201.01 ± 36.86 b

Note: Total canopy deposition under the two flight patterns was compared using Duncan’s multiple range test. Different lowercase letters indicate significant differences at *p* < 0.05.

**Table 4:** Canopy penetration rates of citrus trees under the two flight patterns.

Flight Pattern	Tree A Penetration Rate/%	Tree B Penetration Rate/%	Tree C Penetration Rate/%	Mean/%
Flight pattern 1	13.37	36.68	34.66	28.23 ± 12.98
Flight pattern 2	48.51	26.91	27.04	34.15 ± 12.49

**Table 5:** Rod-position deposition in different areas under the two flight patterns.

Flight Pattern	Rod-Position Area	Sample Size	Mean Deposition/μg	Standard Deviation/μg
Flight pattern 1	Beneath the flight route	6	1810.97	231.16
Flight pattern 2	Beneath the flight route	6	1644.08	303.71
Flight pattern 1	Off-route area	6	268.01	178.71
Flight pattern 2	Off-route area	6	255.59	222.05

## Data Availability

The original contributions presented in this study are included in the article. Further inquiries can be directed to the corresponding author.
